# Diagnostic shift in adolescents with first episode psychosis: findings from the 2-year follow-up of the “Parma Early Psychosis” program

**DOI:** 10.1007/s00127-024-02721-2

**Published:** 2024-06-29

**Authors:** Lorenzo Pelizza, Enrico Plazzi, Emanuela Leuci, Anna Caterina Leucci, Emanuela Quattrone, Silvia Azzali, Simona Pupo, Giuseppina Paulillo, Pietro Pellegrini, Marco Menchetti

**Affiliations:** 1 Department of Biomedical and Neuromotor Sciences, Alma Mater Studiorum Università di Bologna, viale Pepoli 5, Bologna (BO), 40123 Italy; 2 Department of Mental Health and Pathological Addictions, Azienda USL di Parma, largo Palli 1/a, Parma (PR), 43100 Italy; 3 Department of Mental Health and Pathological Addictions, Azienda USL-IRCCS di Reggio Emilia, viale Amendola 2, Reggio Emilia (RE), 42100 Italy; 4 Pain Therapy Service, Department of Medicine and Surgery, Azienda Ospedaliero-Universitaria di Parma, viale Gramsci 14, Parma (PR), 43100 Italy

**Keywords:** Diagnostic stability, First episode psychosis, Adolescence, Early detection, Diagnosis, Follow-up

## Abstract

**Purpose:**

Diagnostic stability for people with First Episode Psychosis (FEP) is essential for treatment, but it remains poorly investigated, especially in adolescents and within a prospective design. The aims of this research were: (a) to examine diagnostic change in Italian adolescents with FEP treated within an “Early Intervention in Psychosis” program during a 2-year follow-up period and (b) to investigate any sociodemographic and clinical predictors at baseline.

**Methods:**

At baseline, 66 adolescents with FEP was recruited. Their primary diagnosis was formulated both at baseline and at the end of follow-up. At presentation, FEP adolescents completed the Health of the Nation Outcome Scales for Children and Adolescents (HoNOSCA). As for diagnostic stability, the Kappa statistic was calculated. The associations of diagnostic change with baseline clinical and sociodemographic features were analyzed using a logistic model with the diagnostic shift as dependent variable. A propensity score was finally calculated based on logistic analysis results.

**Results:**

38 (57.6%) FEP adolescents changed their opening diagnosis. The highest prospective diagnostic stability was for initial diagnosis of schizophrenia (95.4%) and affective spectrum psychoses (75%). Diagnostic instability was high for opening diagnosis of psychosis not otherwise specified, brief psychosis and schizophreniform disorder (100%). The best predictors of diagnostic change were fewer years of education, shorter duration of untreated psychosis and higher baseline levels of psychiatric symptoms.

**Conclusion:**

Diagnostic stability is crucial for treatment and clinical decision making. Addressing instability in FEP diagnoses is an important challenge for future diagnostic development in early psychosis, especially in adolescence.

**Supplementary Information:**

The online version contains supplementary material available at 10.1007/s00127-024-02721-2.

## Introduction

Unlike what happens in most areas of clinical medicine, psychiatric diagnosis, as operationalized in the current international taxonomies (such as the International Classification of Diseases − 11th Edition [ICD-11] and the Diagnostic and Statistical Manual of mental disorders − 5th Edition [DSM-5]) [1, 2], are primarily derived from expert opinion, with the specific aim of improving reliability across clinicians in different cultures [3]. In this respect, the US National Institute of Mental Health specifically commented: “.unlike our definitions of ischemic heart disease, lymphoma or AIDS, DSM-5 diagnoses are based on a consensus about clusters of clinical symptoms, not any biological or laboratory measure” [4]. Since no objective markers or tests are on the horizon [5], clinical psychiatry still remains anchored to the patient’s altered experience, expression and existence, associated with suffering in self and/or others [6]. Therefore, assessing validity and reliability of current diagnostic criteria based on psychopathology is essential especially in patients at their first contact with mental health services and at the onset their clinical trajectory [7].

In particular, the quantification of diagnostic stability/instability of *First Episode Psychosis* (FEP) diagnoses is of paramount practical relevance [8]. Indeed, it is crucial to both ensure diagnostic validity [9] and optimize early interventions in FEP patients [10], especially in light of the limited treatment achievements in the late stages of the disorder [11, 12]. In this respect, as notably observed by Wing and his team in 1967 [13], individuals with psychosis did not necessarily keep the same assigned diagnosis throughout their illness course. Indeed, a meta-analysis on 42 studies examining longitudinal shift in FEP diagnoses [14] showed a high prospective diagnostic instability for psychosis Not Otherwise Specified (NOS) (point estimate = 0.36) and schizophreniform disorder (0.29), despite a high prospective diagnostic stability within schizophrenia spectrum disorders (0.90) and affective psychoses (0.84).

Several studies also investigated potential predictors of *diagnostic shift* in FEP population [15–23]. The main clinical parameters predicting future diagnostic change included long Duration of Untreated Psychosis (DUP), history of substance abuse, long duration of inpatient treatment, experiencing social isolation and higher baseline severity levels of FEP psychopathology. However, in the above mentioned meta-analysis [14], no sociodemographic and clinical predictive factor significantly associated with future diagnostic shift was found.

Furthermore, although no effect for age was observed, most studies included in this meta-analytic research [14] were conducted in mixed adolescent and adult FEP samples. Evidence on diagnostic stability/instability exclusively in FEP adolescent populations is still scarce despite *adolescence* is a relevant sensitive life period in which psychotic symptoms usually emerge for the first time [24]. The specific importance of performing exclusively studies on adolescent with FEP is that different clinical presentation, more incidence of psychosis NOS, and other relevant clinical and diagnostic characteristics could differ from adult patients with early psychotic disorder [7, 14]. In this respect, in a comparative review of diagnostic evaluation in FEP patients, Menezes and Milovan [25] reported that the adolescent population showed greater diagnostic instability than in adults. However, no statistically significant parameter predictive of a diagnostic shift in adolescence was found. In the 2-year prospective follow-up of the “Child and Adolescent First-Episode Psychosis Study” (CAFEPS) (*n* = 83), Castro-Fornieles and co-workers [26] reported a 64% global consistency for all FEP diagnoses, with higher diagnostic stability for bipolar disorder (92%) and schizophrenia spectrum disorders (90%). The lowest values were for psychotic disorder NOS (12%) and brief psychosis (0%). The most common diagnostic shift was to schizophrenia spectrum and bipolar disorders. Independent predictor of change to schizophrenia spectrum disorders was low functioning at baseline.

These findings are substantially in line with what was reported in previous epidemiological investigations [27, 28]. In this respect, in a small sample of 24 Spanish FEP adolescents, Fraguas and colleagues [27] observed a 1-year diagnostic agreement rate of 55%. Schizophrenia had the highest prospective consistency (100%), followed by bipolar disorder (71%), schizoaffective disorder (50%), brief psychosis (50%) and psychosis NOS (16.7%). The most frequent diagnostic shift was to schizophrenia and bipolar disorders. Furthermore, in 68 Swedish FEP inpatient adolescents, Jarbin and von Knorring [28] reported a 72% overall rate of diagnostic consistency across a 1-year follow-up period, with a 100% positive predictive validity from the first episode for schizophrenia, 79% for bipolar disorder and 64% for major depressive disorder.

Although interesting, these findings need to be replicated in FEP adolescent samples with different cultures and languages. Moreover, if the epidemiological study by Castro-Fornieles and co-workers [26] is excluded, empirical evidence on predictors of longitudinal diagnostic shift in adolescents with FEP is lacking. Therefore, the primary *aim* of this longitudinal research was to examine the stability/instability rates of FEP diagnoses in an Italian help-seeking adolescent sample recruited within an “Early Intervention in Psychosis” (EIP) program along a 2-year follow-up period. Additional aim of this study was to investigate any sociodemographic and clinical predictor of diagnostic shift at baseline.

## Methods

### Setting and subjects

Participants of this study were adolescents recruited and treated within the “Parma Early Psychosis” (Pr-EP) program between January 2013 and December 2020. The Pr-EP program is a specialized EIP protocol developed not as a centralized (stand-alone) service, but as a diffuse infrastructure within all adolescent mental healthcare centers of the Parma Department of Mental Health, in Northern Italy [29].

Inclusion criteria for this research were: (a) age 12–18 years; (b) specialist help-seeking request; (c) enrollment in the Pr-EP program; (d) presence of FEP within one of the following DSM-5 diagnoses: schizophrenia (F20), bipolar disorder with psychotic features (F30.2, F31.2, F31.5, F31.6), major depressive disorder with psychotic features (F32.3, F33.3), delusional disorder (F22), brief psychotic disorder (F23), schizophreniform disorder (F25), and psychotic disorder NOS (F29) [1]; and (e) a DUP of < 2 years. The DUP was defined as the time interval between the onset of psychotic symptoms and the first antipsychotic intake [30]. A DUP of < 2 years was specifically selected because it is the usual limit to provide effective specialized interventions within the EIP paradigm [31].

Exclusion criteria were: (a) past DSM-5 affective or non-affective psychotic episode; (b) past exposure to antipsychotic medication; (c) known intellectual disability (i.e., Intelligent Quotient < 70); and (d) neurological disease or any other medical disorder presenting with psychiatric symptoms. In this investigation, past exposure to antipsychotic drug (i.e., at any dosage and however before the Pr-EP enrollment) was considered as “functional equivalent” of a past psychotic episode [32, 33]. Indeed, in line with this conceptualization, the traditional EIP paradigm psychometrically defined the “psychosis threshold” as essentially that at which antipsychotic medication would probably be commenced in the common clinical practice [34].

All participants and their parents agreed to participate to this investigation and gave their written informed consent prior to their inclusion in the study. Local relevant ethical approval was obtained for the research (AVEN Ethics Committee protocol n. 36,102/2019). This investigation was also conducted in accordance with the Code of Ethics of the World Medical Association (Declaration of Helsinki) for experiments including humans.

### Assessment

For all participants, a sociodemographic/clinical chart (including information on gender, age at entry, nationality, years of education, employment status, past specialist contact, current substance abuse and DUP) was filled in at entry. The DSM-5 diagnoses were formulated two times, at baseline and at the end of the 2-year follow-up period by at least two trained Pr-EP team members, using the “Structured Clinical Interview for DSM-5 mental disorder [35]. Although created for adult population, we used this instrument for examining the DSM-5 diagnostic criteria for mental disorders in our FEP participants, following a practice commonly used in mixed adolescent and young adult FEP populations [7, 24]. Specifically, we focused on the psychosis part of the interview, also asking parents/caregivers to enrich collected information, especially in the youngest ages.

The presence of FEP at baseline was then confirmed using psychometric criteria for psychosis threshold as defined in the “Comprehensive Assessment of At-Risk Mental States” (CAARMS), approved Italian version [36]. Specifically, the definition of CAARMS criteria was used.

The clinical assessment included the Health of the Nation Outcome Scale for Children and Adolescent (HoNOSCA) [37], the Positive And Negative Syndrome Scale (PANSS) [38] and the Global Assessment of Functioning (GAF) scale [1]. These instruments were completed by trained Pr-EP team members (i.e., child and adolescent psychiatrists and psychologists) at baseline. Regular scoring workshops and supervision sessions ensured inter-rater reliability of the scales [39].

The HoNOSCA is a clinical interview specifically developed to assess social and clinical outcomes in children and adolescents with severe mental illness. It includes 13 items rated 0–4, which are added together to create a total score (0–52). Currently, it is frequently used also in young Italian FEP populations [40]. As proposed by Speak and Muncer [41], we considered the following 4 main outcome domains: “Psychiatric Symptoms”, “Impairment”, “Social Problems” and “Behavioral Problems”.

The PANSS is a clinical interview specifically designed to assess psychosis psychopathology and frequently used also in Italian adolescent FEP samples [42]. The patient is rated from 1 to 7 on its 30 different items, which are added together to create a total score (30–210). As proposed by Shafer and Dazzi [43], we considered 5 main clinical dimensions: “Negative Symptoms”, “Affect” (“Depression/Anxiety”), “Positive Symptoms”, “Disorganization” and “Resistance/Excitement-Activity”.

The GAF is a widely used scale to assess daily functioning in individuals with psychosis. It has been frequently administered also in Italian adolescents with FEP [44]. Its score ranges from 0 to 100, with 100 representing superior daily functioning.

### Procedures and statistical strategy

FEP adolescents with Diagnostic Shift (DS) across the follow-up period were included in the FEP/DS + subgroup, while FEP individuals without DS were grouped in the FEF/DS- subsample.

The Pr-EP program provided a 2-year comprehensive treatment package including psychopharmacological therapy and multi-element psychosocial intervention (combining individual psychotherapy based on cognitive-behavioral principles, psychoeducational sessions for family members and a recovery-oriented case management) in accordance with the current EIP guidelines [45].

Data were analyzed using the Statistical Package for Social Science (SPSS) for Windows, version 15.0 [46], and the R project for statistical computing (CRAN package) [47] (R Core Team, 2022). Statistical analyses were two-tailed, with a significance level set at 0.05.

For an initial assessment of diagnostic stability, the kappa statistic was calculated [48]. This is a widely used method to measure the concordance between opening and final diagnoses. The k values range from − 1 to + 1. A score of + 1 indicates perfect agreement, while a value of -1 indicates perfect discordance. Specifically, k values of 0.10–0.20 indicated slight agreement, k values of 0.21–0.40 fair agreement, k values of 0.41–0.60 moderate agreement, k values of 0.61–0.80 substantial agreement, and k values of 0.81–0.99 near perfect agreement [48]. A logistic regression model was then estimated using a “dummy” coding the diagnostic shift as dependent parameter and baseline sociodemographic and clinical features as independent variables. Specifically, all sociodemographic and clinical variables collected at baseline were introduced in the model.

Furthermore, using the theoretical assumptions of the Propensity Score (PS) [49], a propensity measure for DS was constructed on logistic regression analysis results. The PS is the estimated probability of developing a diagnostic shift. Each participant was assigned by a PS score ranged from 0 to 1 (with 0 indicating no propensity for diagnostic change and 1 indicating a very high propensity for diagnostic shift). In this respect, being computed considering all the collected baseline sociodemographic and clinical characteristics, the PS model was useful to identify those drivers influencing the likelihood of experiencing diagnostic shift along the follow-up. Indeed, the PS allowed the risk of each FEP participant to be assessed, and (using its quartiles) allowed the total sample to be divided into bands of longitudinal propensity to diagnostic change (starting from the baseline parameters introduced into the initial model). Based on the estimated PS, the total sample was then stratified into 4 main subgroups using median and quartile values as cut-offs (subgroup I = PS ≤ first quartile; subgroup II = first quartile < PS ≤ median; subgroup III = median < PS ≤ third quartiles; subgroup IV = PS > third quartile). For each group, diagnostic flows were described and kappa statistics were then calculated.

## Results

Sixty-six FEP adolescents were recruited within the Pr-EP program between January 2013 and December 2020 (34 [51.5%] males; mean age = 17.09 ± 1.36 years). After the 2-year follow-up period, 38 (57.6%) FEP participants changed their initial diagnosis and were included in the FEP/DS + subgroup. The remaining 28 subjects were grouped in the FEP/DS- subsample. Baseline clinical and sociodemographic features of the FEP total group and the two subgroups are shown in the Table 1.

**Table 1:** Baseline sociodemographic and clinical characteristics of the FEP total sample and the two subgroups, and results from the logistic regression model for PS estimate

Variable	FEP total sample (*n* = 66)	FEP/DS- (*n* = 28)	FEP/DS+ (*n* = 38)	B	OR	*p*	95% CI for OR Lower Upper	
Gender (female) Age at entry NEET Nationality (Italian) Education (in years) Previous specialist contact DUP (in months) Substance abuse at entry Baseline AP prescription Baseline AD prescription Baseline BDZ prescription Baseline MS prescription	48.5% 17.09 (1.36) 15.1% 75.8% 10.86 (1.48) 48.5% 10.32 (10.26) 34.8% 15.1% 48.9% 28.8% 15.1%	42.9% 17.04 (1.29) 17.9% 75.0% 11.00 (1.59) 50.0% 13.87 (10.21) 28.6% 10.7% 42.9% 32.1% 10.7%	57.9% 17.13 (1.42) 13.2% 76.3% 10.76 (1.40) 47.4% 7.70 (9.60) 39.5% 18.4% 57.9% 26.3% 5.3%	0.072 − 0.045 0.142 0.044 − 0.003 − 0.305 − 0.063 0.412 0.645 0.301 0.263 − 0.667	0.931 0.956 1.152 0.957 1.003 0.737 0.939 1.510 1.906 1.351 1.301 0.513	0.718 0.060 0.980 0.800 **0.020** 0.560 **0.010** 0.210 0.220 0.390 0.180 0.760	0.631 0.927 0.719 0.616 0.940 0.505 0.918 1.028 1.076 0.838 0.881 0.293	1.374 0.986 1.846 1.485 1.071 1.076 0.961 2.217 3.375 2.179 1.921 0.898
**Baseline** ***HoNOSCA*** **scores**								
Total score Psychiatric Symptoms Social Problems Behavioral Problems Impairment	23.58 (7.89) 10.24 (3.44) 6.92 (3.46) 3.48 (2.46) 2.46 (2.92)	23.43 (8.08) 9.71 (3.82) 7.07 (3.35) 3.46 (2.59) 3.18 (2.20)	23.70 (7.84) 10.63 (3.13) 6.82 (3.57) 3.50 (2.40) 2.73 (1.84)	0.015 0.053 0.018 0.039 − 0.005	1.015 1.074 1.018 0.730 0.995	0.266 **0.020** 0.620 0.180 0.260	0.993 0.995 0.970 0.936 0.910	1.038 1.116 1.069 1.022 1.088
**Baseline** ***PANSS*** **scores**								
Total score Affect Disorganization Resistance/Excitement Negative Symptoms Positive Symptoms	87.69 (20.70) 18.21 (5.70) 19.29 (7.19) 8.81 (4.20) 22.98 (7.98) 17.63 (5.32)	84.43 (18.84) 17.83 (5.84) 18.13 (6.70) 8.33 (4.49) 23.08 (8.19) 16.38 (5.04)	91.63 (22.63) 18.68 (5.63) 20.68 (7.68) 9.42 (3.85) 22.84 (7.94) 19.21 (5.37)	0.002 0.006 0.009 − 0.008 0.004 − 0.014	1.000 1.006 1.009 0.992 1.004 0.986	0.261 0.287 0.827 0.492 0.321 0.763	0.990 0.967 0.978 0.945 0.977 0.948	1.010 1.048 1.040 1.041 1.031 1.026
**Baseline** ***GAF*** **score**	44.49 (11.08)	43.32 (12.11)	45.42 (10.36)	0.010	1.010	0.535	0.988	1.033

Note. FEP = First Episode Psychosis; DS = Diagnostic Shift; FEP/DS- = FEP adolescents without DS; FEP/DS + = FEP adolescents with DS; PS = Propensity Score; B = Regression coefficient; OR = Odds Ratio; p = statistical significance; CI = 95% Confidence Intervals for OR; NEET = Not in Education, Employment or Training; DUP = Duration of Untreated Psychosis; AP = Antipsychotic; AD = Antidepressant; BDZ = Benzodiazepine; MS = Mood Stabilizer; HoNOSCA = Health of the Nation Outcome Scales for Children and Adolescents; PANSS = Positive And Negative Syndrome Scale; GAF = Global Assessment of Functioning. Percentages and mean (standard deviation) values are reported

Diagnostic workflows (from baseline diagnosis to any final diagnoses across the follow-up period) are shown in the Table 2. Specifically, psychotic disorder NOS, brief psychosis and schizophreniform disorder had the highest diagnostic shift rates (100%), followed by schizoaffective disorder (66.7%). Schizophrenia is the most longitudinally stable diagnosis (DS rate = 4.5%), followed by affective psychosis (25%). A kappa value of 0.35 indicated medium to low concordance between opening and final diagnoses.

**Table 2:** Distribution of FEP adolescents with DS by diagnostic categories at baseline and after the 2 years of follow-up (*n* = 66)

Final diagnosis	Baseline diagnosis							
	Schizophrenia	Affective Psychosis	Substance-Induced Psychosis	Brief Psychotic disorder	Psychosis NOS	Schizoaffective Disorder	Schizophreniform Disorder	Total
Schizophrenia	21	2		4	5	1	1	34
Affective Psychosis		6		3	7	1	6	23
Substance-Induced Psychosis					1			1
Schizoaffective Disorder	1					1		2
Schizotypal Personality Disorder				1	1		2	4
Borderline Personality Disorder			1	1				2
**Total**	22	8	1	9	14	3	9	66
% in the total sample	33.3%	12.1%	1.52%	13.6%	21.2%	4.5%	13.6%	100.0%
DS (%)	4.5%	25.0%	100.0%	100.0%	100.0%	66.7%	100.0%	57.6%
OP	0.42							
EP	0.11							
Kappa	0.35							

Note. FEP = First Episode Psychosis; DS = Diagnostic Shift; NOS = Not Otherwise Specified; OP = Proportion of Observed concordance between initial and final diagnoses; EP = Expected Proportion of concordance between initial and final diagnoses; DS (%) was calculated as “x_d_/n_d_” (where “d” indicated the initial diagnosis, “x” the number of FEP patients with diagnostic shift and “n” the number of FEP patients with initial “d” diagnosis). Frequencies, percentages and kappa values are reported

Table 3 showed that 95.4% FEP adolescents with initial schizophrenia confirmed their diagnosis at the end of our follow-up. Similarly, 75% FEP participants with affective psychosis at entry maintained their diagnosis in the final diagnostic reformulation. Within adolescents with brief psychotic disorder at baseline, 44.4% shifted to schizophrenia and 33.3% to affective psychosis. Moreover, 50% psychotic disorder NOS at entry shifted to affective psychosis during the follow-up period and 35.7% to schizophrenia. Within FEP patients with schizophreniform disorder at baseline, 66.7% shifted to affective psychosis and 22.2% to schizotypal personality disorder. Finally, schizophrenia and affective psychosis (33.3%) were the main diagnostic shifts across the follow-up in adolescents with schizoaffective disorder at presentation.

**Table 3:** Proportion of FEP adolescents with diagnostic shift by baseline diagnosis (*n* = 66)

	Schizophrenia				Affective Psychosis				Substance-Induced Psychosis		
	Percentage	Jeffrey CI (lower).	Jeffrey CI (upper)		Percentage	Jeffrey CI (lower)	Jeffrey CI (upper)		Percentage	Jeffrey CI (lower)	Jeffrey CI (upper)
Schizophrenia	95.45	77.16	99.88	Schizophrenia	25.00	3.19	65.09	Borderline Personality Disorder	100.00	2.50	100.00
Schizoaffective Disorder	4.55	0.12	22.84	Affective Psychosis	75.00	34.91	96.81				
	Brief Psychotic Disorder				Psychosis NOS				Schizoaffective Disorder		
	Percentage	Jeffrey CI (lower)	Jeffrey CI (upper)		Percentage	Jeffrey CI (lower)	Jeffrey CI (upper)		Percentage	Jeffrey CI (lower)	Jeffrey CI (upper)
Schizophrenia	44.44	13.70	78.80	Schizophrenia	35.71	12.76	64.86	Schizophrenia	33.33	0.84	90.57
Affective Psychosis	33.33	7.49	70.07	Affective Psychosis	50.00	23.04	76.96	Affective Psychosis	33.33	0.84	90.57
Schizotypal Personality Disorder	11.11	0.28	0.28	Substance-Induced Psychosis	7.14	0.18	33.87	Schizoaffective Disorder	33.33	0.84	90.57
Borderline Personality Disorder	11.11	48.25	48.25	Schizotypal Personality Disorder	7.14	0.18	33.87				
	Schizophreniform Disorder										
	Percentage	Jeffrey CI (lower)	Jeffrey CI (upper)								
Schizophrenia	11.11	0.28	48.25								
Affective Psychosis	66.67	29.93	92.51								
Schizotypal Personality Disorder	22.22	2.81	60.01								

Note. FEP = First Episode Psychosis; NOS = Not Otherwise Specified; CI = Confidence Intervals. Percentages are reported

Table 1 also shows the estimated odds ratios from our logistic regression analysis. A significant predictive role of *DUP* for longitudinal diagnostic change was observed (i.e., as the DUP increased, the likelihood of a shift in psychosis diagnosis decreased). Another robust predictor of diagnostic shift was the *educational level* (i.e., the fewer years of education, the higher the likelihood of change in psychosis diagnosis). Finally, the likelihood of a shift in diagnosis increased together with increasing in baseline severity levels of *HoNOSCA “Psychiatric Symptoms”* domain score.

The mean of the *PS* obtained from the model estimated on the FEP total sample was 0.58 ± 0.19 (minimum value = 0.06, maximum value = 0.84). The median of the PS was 0.63 with an interquartile range between 0.44 and 0.72. These quartiles allowed us to identify 4 main FEP subgroups characterized by an increasing longitudinal propensity to diagnostic shift (Table 4). The first was the most stable subgroup and consisted of only 9 FEP adolescents with a prevalent diagnosis of schizophrenia (44.4%). In the second subgroup (*n* = 12), schizophrenia remained the modal opening diagnosis (50%), but in comparison with the subgroup I, a significantly higher percentage of affective psychosis was found (25%). The proportion of FEP adolescents with an initial diagnosis of schizophrenia (35%) was even lower in the subgroup III (*n* = 20), although it was the modal diagnosis. In this subgroup, the diagnosis of psychosis NOS was also high (30%) and the proportions of FEP individuals with schizophreniform disorder increased (15%). Finally, the subgroup IV (*n* = 25) had the highest propensity score and the proportions of brief psychotic disorder (28%) grew further, so becoming the modal diagnosis (followed by schizophreniform disorder [20%]). Although k values calculated for each subgroup were all moderate, they confirmed that the subgroup I was the one with the highest diagnostic concordance (k = 0.39) and the subgroup IV was the one with the lowest concordance (k = 0.21).

**Table 4:** Baseline diagnosis distribution in the 4 subgroups identified through the propensity score in the FEP total sample (*n* = 66)

	Subgroup I (*n* = 9)	Subgroup II (*n* = 12)	Subgroup III (*n* = 20)	Group IV (*n* = 25)	I vs. II	I vs. III	I vs. IV	II vs. III	II vs. IV	III vs. IV
Kappa	0.39	0.31	0.24	0.21						
Schizophrenia	44.44	50.00	35.00	20.00		^*^	^***^	^**^	^***^	^***^
Affective psychosis	11.11	25.00	10.00	8.00	^***^		^*^	^***^	^***^	
Substance-induced psychosis	11.11	0.00	0	0.00	^***^	^***^	^***^	^***^	^***^	^***^
Brief psychosis	0.00	0.00	10.00	28.00	^***^	^***^	^***^	^***^	^***^	^***^
Psychosis NOS	22.22	16.67	30.00	16.00						
Schizoaffective disorder	11.11	0.00	0.00	8.00	^***^	^***^	^***^	^***^	^***^	
Schizophreniform disorder	0.00	8.33	15.00	20.00	^***^	^***^	^***^	^***^	^***^	^***^

Note. FEP = First Episode Psychosis; NOS = Not Otherwise Specified. Kappa values and percentages are reported. ^***^p < 0.001, ^**^p < 0.01; ^*^p < 0.05

## Discussion

The results of this research first support a wide *construct variability* across prospective diagnostic stability of different FEP diagnoses, also in adolescent patients. In this respect, according to what was reported in previous studies with comparable sample size [26–28], we observed high diagnostic stability for schizophrenia and affective spectrum psychosis over time. This longitudinal stability is crucial for all international agencies licensing the use of medications in mental disorders (e.g., the European Medicines Agency [EMA] in Europe). As an example, the EMA approved the use of paliperidone exclusively for the treatment of schizophrenia spectrum disorder, risperidone for schizophrenia and manic episode in bipolar disorder, and haloperidol for psychotic disorders in general [50]. However, the off-label prescription of these antipsychotic medications still remains very common in patients with psychosis, but it is not without health-economic and legal implications [51].

Anyway, it is also important to point out that not all aspects of *diagnostic shift* are negative at the psychosis onset. Indeed, although diagnostic stability is a key criterion for the “nosological” validity of most FEP categories, some psychiatric diagnoses are formulated “a priori” on expected diagnostic uncertainty at entry or on insufficient information available at baseline for a specific psychotic disorder [52]. Moreover, as it is no easy for a family and for an adolescent boy or girl to assume a diagnosis of schizophrenia, clinicians had to be very cautious. Indeed, having a false diagnosis of these magnitude could affect to patient and family, and could become on the wrong treatment. In this respect, some authors intend these unstable diagnostic categories (especially psychosis NOS, schizophreniform disorder and brief psychotic disorder) as “place-holders” [14], in which frequent diagnostic shifts are to be expected. In this investigation, we found that all initial cases of brief psychosis, psychosis NOS and schizophreniform disorder changed their diagnostic status during the 2-year follow-up period. In our opinion, repeated, in-depth diagnostic assessments of initial unstable and/or clinically undefined FEP categories allow us to better explore and describe the patient’s subjective suffering, to enhance clinical adherence to current guidelines indicating specific (evidence-based) treatments for different psychotic disorders [53], and to increase therapeutic alliance and motivation to care [54].

Diagnostic shift from schizophrenia to other psychotic disorders was vary uncommon (4.5%) in our adolescent FEP population, while 2 (25%) out of 8 FEP patients with initial affective psychosis shifted towards schizophrenia. These findings are substantially in line with what was reported in previous studies on adolescent FEP populations with comparable sample size [26–28], and are of direct clinical relevance for mental health professionals who have to follow the differential guidelines for early affective spectrum psychoses vs. schizophrenia. Indeed, this confirms the importance of correctly formulating the diagnosis as soon as the adolescent enters EIP services [55], because the opening FEP diagnosis, if incorrect, may hinder the indicated clinical care not only in terms of specific psychosocial interventions and pharmacotherapies, but also in the descriptions provided to newly diagnosed individuals and their family members as to what lies ahead [56].

Moreover, we observed that diagnostic shift was particularly frequent to schizophrenia (44.4% of initial brief psychosis and 35.7% of initial psychotic disorder NOS) and affective spectrum psychosis (66.7% of initial schizophreniform disorder, 50% of initial psychosis NOS and 33.3% of initial brief psychosis), followed by schizotypal personality disorder (22.2% of initial schizophreniform disorder and 11.1% of initial brief psychotic disorder). These results are substantially in line with what was reported in previous comparable studies [26, 27], although with a lower global diagnostic stability in our research (35% vs. 55-63.9%). This difference may be attributable to different follow-up lengths (1 vs. 2 years). Overall considered, these findings suggest that a relevant number of FEP adolescents may receive a “provisional” diagnosis at baseline [25], and that careful re-assessments and monitoring of FEP individuals (especially those with initial less defined, unstable and/or remitting psychosis diagnosis) are therefore crucial [57]. Indeed, having a first diagnosis of brief psychosis or psychotic disorder NOS is probably linked to the time of symptoms presentation (e.g., DUP length or other longitudinal variables), and diagnosis of schizophrenia is often not possible at a first moment for some FEP patients.

Additional aim of this study was to identify any *predictor of diagnostic instability* so as to detect specific baseline characteristics of those FEP patients who may be misdiagnosed at entry. In this investigation, a *shorter DUP*, *fewer years of education* and higher severity levels in *psychiatric symptoms* at entry were statistically significant predictive factors for a prospective diagnostic shift in our FEP adolescents, especially in the subgroup IV and from less clinically and/or temporally defined psychosis diagnoses towards more stable FEP categories (e.g., schizophrenia and affective spectrum psychosis). These baseline characteristics should thus be carefully monitored in FEP adolescents at the recruitment in EIP services because of their crucial role as putative indicators of greater diagnostic severity over time. To our knowledge, no other study using PS measures was published in the literature to date. Therefore, precisely because the PS methods allowed us to better investigate the predictive factors of diagnostic shift in FEP patients over time, future research with the same statistical strategy for comparing our PS findings are needed.

As for predictors of longitudinal diagnostic shift in adolescents with FEP, empirical evidence is poor and inconsistent. Castro-Fornieles and co-workers [26] found that independent predictive factors of change to schizophrenia spectrum disorders in a sample of 83 youngsters (aged 9–17 years) recruited within the 2-year follow-up of the “Child and Adolescent First-Episode Psychosis Study” (CAFEPS) in Spain were lower scores on the “Children’s Global Assessment Scale” (C-GAS) and the “Hamilton Depression Rating Scale (HDRS). In a comparative review of diagnostic evolution and predictive parameters in adolescent versus adult FEP patients, Menezes and Milovan [25] observed a greater diagnostic instability in the adolescent population than in adults, and reported that premorbid adjustment in adolescents and GAF both before and after FEP in adolescents and adults were the best predictors of diagnosis. In this respect, comparing adolescent and adult FEP patients enrolled in the Pr-EP program, we observed comparable, modest diagnosis concordance rates (k values = 0.34 vs. 0.35), but relevant differences in predictors of prospective diagnostic shift (see Supplementary Materials [Table S1] for details). Indeed, our FEP adults shared a shorter DUP, but differed for the predictive role of previous specialist contact in child/adolescent mental health services and higher prescription rates of both antidepressant and benzodiazepine at entry. In our FEP adults, no role in longitudinal diagnostic change was played by greater severity levels of psychiatric symptoms at presentation.

In mixed adolescent and young adult FEP populations, Schimmelmann and colleagues [58] reported that the best predictors of shift from schizophreniform disorder to schizophrenia or schizoaffective disorder in 786 FEP individuals enrolled within the “Early Psychosis Prevention and Intervention Centre” (EPPIC) in Australia were a lower premorbid functioning and higher scores on the “Clinical Global Impression” (CGI) at entry. In a sample of 301 FEP subjects from four national healthcare sectors in Denmark and Norway, Haahr and co-workers [59] differently found that features discriminating schizophreniform individuals developing schizophrenia at 1 year were longer DUP, less severe general psychotic symptoms, male gender and poor premorbid functioning. In 150 South-Korean FEP individuals, Kim and colleagues [18] observed that female gender, good premorbid functioning, shorter DUP and higher severity levels in positive and manic symptoms at baseline were strong predictors of diagnostic change from non-affective psychosis to bipolar disorder. Finally, meta-analytic results by Fusar-Poli and co-workers [14] showed that there was greater diagnostic stability when the initial diagnosis was formulated in inpatient units compared to mixed medical setting. In this respect, these authors concluded that there were not enough studies reporting on predictors of diagnostic stability in FEP patients (especially in adolescence), and that their meta-analysis carried over limitations of these original investigations.

### Limitations

Several limitations of this study are also to be acknowledged. A first weakness was the small sample size. This may have limited the strength and accuracy of our statistical analysis results. Therefore, future investigations to replicate our findings in larger FEP adolescent populations are needed.

Second, our follow-up period was relevant, but limited to 2 years. Longer perspective research to confirm and extend our results is thus needed, especially those aiming at investigating relevant predictors of longitudinal diagnostic change.

Finally, we used the DSM-5 diagnostic criteria for mental disorders in our FEP adolescents, following a practice commonly used in mixed adolescent and young adult FEP populations [60]. This may be of particular concern when formulating a diagnosis of personality disorders, which would require the age of > 18 years. Future studies specifically examining adolescent FEP samples with more indicated clinical interviews (such as the Kiddie schedule for mental disorders) [61] are thus needed.

## Conclusions

The results of this investigation support a high prospective diagnostic stability for FEP adolescents with initial diagnosis of schizophrenia and affective spectrum psychoses. However, diagnostic stability overall seems to be lower than what was previously reported, especially for an opening diagnosis of brief psychotic disorder, schizophreniform disorder and psychosis NOS. In this research, most of diagnostic shift were towards schizophrenia, affective psychosis and schizotypal personality disorders.

The results of this study also showed that the best predictors of longitudinal diagnostic change in our FEP adolescents are a shorter DUP, fewer years of education and higher baseline severity levels in psychiatric symptoms. As diagnostic stability is important for both caretakers and patients and offers general guidance for clinical decision making and the development of treatment guidelines [62], addressing instability in FEP diagnosis and its potential predictors at baseline is an important challenge for future diagnostic evolution of early psychosis, especially in adolescence.

## Electronic supplementary material

Below is the link to the electronic supplementary material.

Supplementary Material 1

## Data Availability

The data that support the findings of this investigation are available on reasonable request from the corresponding author. The data are not publicly available due to privacy/ethical restrictions.
