# Supplementary material for: Diagnostic shift in adolescents with first episode psychosis: findings from the 2-year follow-up of the “Parma Early Psychosis” program
Source: Soc Psychiatry Psychiatr Epidemiol. 2024 Jun 29;60(2):375–85. doi: 10.1007/s00127-024-02721-2 (PMC11839870; doi:10.1007/s00127-024-02721-2)
Supplement: Supplementary file 1 — Supplementary Material 1 [file 127_2024_2721_MOESM1_ESM.docx]

Table S1. Results from logistic regression model for PS estimate.

|  | **Adolescent sample**  **(n = 66)** | | **Adults sample**  **(n = 375)** | |
| --- | --- | --- | --- | --- |
|  | **OR** | **P-value** | **OR** | **P-value** |
| Age | .956 | 0.06 | 0.99 | 0.65 |
| Female | .931 | 0.08 | 1.03 | 0.82 |
| Education (in years) | 1.003 | 0.02 | 1.05 | 0.26 |
| Unemployed | .756 | 0.98 | 1.19 | 0.29 |
| Student | .564 | 0.99 | 0.91 | 0.70 |
| Italian patients | .957 | 0.80 | 0.91 | 0.49 |
| DUP (in months) | .939 | 0.01 | 0.93 | <0.0001 |
| Previous specialist contact | .737 | 0.56 | 1.85 | 0.01 |
| Drug abuse at entry | 1.510 | 0.21 | 1.10 | 0.48 |
| Baseline Antidepressant prescription | 1.351 | 0.39 | 0.80 | 0.15 |
| Baseline Benzodiazepine prescription | .513 | 0.18 | 0.81 | 0.09 |
| Baseline Mood Stabilizer prescription | 1.301 | 0.76 | 0.75 | 0.09 |
| HoNOSCA/HoNOS “Psychiatric Symptoms” score | 1.074 | 0.02 | 1.02 | 0.60 |
| HoNOSCA/HoNOS “Social Problems” score | 1.018 | 0.62 | 1.01 | 0.89 |
| HoNOSCA/HoNOS “Behavioral Problems” score | .730 | 0.18 | 1.02 | 0.75 |
| HoNOSCA/HoNOS “Impairment” score | .995 | 0.26 | 1.02 | 0.79 |

Note – PS = Propensity score; DUP = Duration of untreated psychosis; CAMHS = Child/Adolescent Mental Health Services; HoNOSCA = Health of the Nation Outcome Scales for Children and Adolescents; HoNOS = Health of the Nation Outcome Scale.
